# Leukocyte Esterase Activity in Vaginal Fluid of Pregnant and Non-Pregnant Women With Vaginitis/Vaginosis and in Controls

**DOI:** 10.1155/S1064744903000036

**Published:** 2003

**Authors:** Per-Anders Mårdh, Natalia Novikova, Ola Niklasson, Zoltan Bekassy, Lennart Skude

**Affiliations:** 1 Department of Obstetrics and Gynecology Lund University Hospital Lund SE 22185 Sweden; 2 Department of Clinical Chemistry County Hospital Halmstad Sweden

## Abstract

*Objectives:* To determine the leukocyte esterase (LE) activity in vaginal lavage fluid of women with acute and recurrent vulvovaginal candidosis (VVC and RVVC respectively), bacterial vaginosis (BV), and in pregnant and non-pregnant women without evidence of the three conditions. Also to compare the result of LE tests in women consulting at different weeks in the cycle and trimesters of pregnancy.The LE activity was correlated to vaginal pH, number of inflammatory cells in stained vaginal smears, type of predominating vaginal bacteria and presence of yeast morphotypes.

*Methods:* One hundred and thirteen women with a history of RVVC, i.e. with at least four attacks of the condition during the previous year and who had consulted with an assumed new attack of the condition, were studied. Furthermore, we studied 16 women with VVC, 15 women with BV, and 27 women attending for control of cytological abnormalities, who all presented without evidence of either vaginitis or vaginosis. Finally, 73 pregnant women were investigated. The LE activity in vaginal fluid during different weeks in the cycle of 53 of the women was measured.

*Results:* In the non-pregnant women, an increased LE activity was found in 96, 88, 73 and 56% of those with RVVC, VVC and BV and in the non-VVC/BV cases, respectively. In 73% of pregnant women in the second trimester, and 76% of those in the third, the LE test was positive. In all groups of non-pregnant women tested, the LE activity correlated with the number of leukocytes in vaginal smears, but it did not in those who were pregnant. There was no correlation between LE activity and week in cycle. The vaginal pH showed no correlation to LE activity in any of the groups studied.

*Conclusions:* The use of commercial LE dipsticks has a limited value in the differential diagnosis of RVVC, VVCand BV. There is no correlation between the LE activity in vaginal secretion on one hand and vaginal pH, week in the menstrual cycle and trimester in pregnancy on the other. Women with BV often have signs of inflammation as evidenced by a positive LE test and inflammatory cells in genital smears.


*Infect Dis Obstet Gynecol 2003;11:19–26*

### Leukocyte esterase activity in vaginal fluid of pregnant and non-pregnant women with vaginitis/vaginosis and in controls

**Per-Anders Mårdh[1], Natalia Novikova[1], Ola Niklasson[1], Zoltan Bekassy[1] and Lennart Skude[2]**

[1]*Department of Obstetrics and Gynecology, University Hospital, Lund and*
[2]*Department of Clinical Chemistry, County Hospital, Halmstad, Sweden*

***Objectives:*** To determine the leukocyte esterase (LE) activity in vaginal lavage fluid of women with acute and recurrent vulvovaginal candidosis (VVC and RVVC respectively), bacterial vaginosis (BV), and in pregnant and non-pregnant women without evidence of the three conditions. Also to compare the result of LE tests in women consulting at different weeks in the cycle and trimesters of pregnancy. The LE activity was correlated to vaginal pH, number of inflammatory cells in stained vaginal smears, type of predominating vaginal bacteria and presence of yeast morphotypes.

***Methods:*** One hundred and thirteen women with a history of RVVC, i.e. with at least four attacks of the condition during the previous year and who had consulted with an assumed new attack of the condition, were studied. Furthermore, we studied 16 women with VVC, 15 women with BV, and 27 women attending for control of cytological abnormalities, who all presented without evidence of either vaginitis or vaginosis. Finally, 73 pregnant women were investigated. The LE activity in vaginal fluid during different weeks in the cycle of 53 of the women was measured.

***Results:*** In the non-pregnant women, an increased LE activity was found in 96, 88, 73 and 56% of those with RVVC, VVC and BV and in the non-VVC/BV cases, respectively. In 73% of pregnant women in the second trimester, and 76% of those in the third, the LE test was positive. In all groups of non-pregnant women tested, the LE activity correlated with the number of leukocytes in vaginal smears, but it did not in those who were pregnant. There was no correlation between LE activity and week in cycle. The vaginal pH showed no correlation to LE activity in any of the groups studied.

***Conclusions:*** The use of commercial LE dipsticks has a limited value in the differential diagnosis of RVVC, VVC and BV. There is no correlation between the LE activity in vaginal secretion on one hand and vaginal pH, week in the menstrual cycle and trimester in pregnancy on the other. Women with BV often have signs of inflammation as evidenced by a positive LE test and inflammatory cells in genital smears.

Key words: LEUKOCYTE ESTERASE; VULVOVAGINAL CANDIDOSIS; RECURRENT VULVOVAGINAL CANDIDOSIS; BACTERIAL VAGINOSIS; PREGNANCY

If the office diagnosis of vaginitis/vaginosis is only based on history and clinical examination, the accuracy will be unacceptably low[1–3]. To diagnose inflammatory reactions in the lower female genital tract, for example of vaginitis, microscopy of vaginal fluid for detection of granulocytes has been the standard procedure. Bacterial vaginosis (BV) has been considered to be an –osis, that is, a

Correspondence to: Per-Anders Mårdh, Department of Obstetrics and Gynecology, Lund University Hospital, SE 22185 Lund, Sweden. E-mail: per-anders.mardh@simrishamn.se

© 2003 The Parthenon Publishing Group 

non-inflammatory condition. Amsel's criteria[4], which do not include the presence of inflammatory cells in vaginal fluid, have generally been employed to diagnose BV.

Leukocyte esterase (LE) tests have been launched as one of several chemical tests on dipsticks employed in the diagnosis of urinary tract infections (UTI). Contradictory results of the diagnostic value of LE tests have been reported[5,6]. A number of studies have reported on the use of an LE dipstick test of urine to diagnose urthritis in males[7,8], while only single studies have used such tests in the diagnosis of female genital infections[9–11].

The LE test is based on detection of an enzyme that hydrolyses indoxyl carboxylic acid ester to indoxyl, which in turn reacts with a diazonium salt to produce a purple colour[12].

The present study was aimed to determine the LE activity in vaginal secretion of pregnant and non-pregnant women with and without evidence of vaginitis and vaginosis. Samples collected at different weeks of the menstrual cycle and at the second and third trimesters of the gestational period were studied. We also studied any correlation between the LE test results and the number of inflammatory cells in vaginal smears.

### SUBJECTS AND METHODS

### Leukocyte esterase tests and pH determinations

The LE activity was measured by a commercial kit (Ecur[4]-Test, Boehringer–Mannheim, later renamed Combur-4 and sold by Roche, Basle). One drop (approximately 0.3 μl) of vaginal lavage fluid was mixed with 3 ml of 0.1M TRIS HCl buffer, pH 7.3 or with physiological saline. The tests were performed within a couple of minutes of sampling. According to the package insert, color changes of grades 1, 2 and 3 correspond to 10–25, approximately 75, and approximately 500 granulocytes/μl, respectively.

Another 1–2 drops of the vaginal lavage were placed on an indicator strip for pH determination (Merck, Darmstadt, Germany). Vaginal samples were tested for LE activity within some minutes as well as after 6 hours' storage[2] at room temperature in TRIS buffer and in physiological saline.

The LE activity was correlated with the predominating bacterial flora, independent of the presence of candida morphotypes. The presence of extremely elongated Gram-positive rods (of lactobacilli morphotype) was also noted.

### Patients with a history of recurrent vulvovaginal candidosis

One of the cohorts studied comprised 113 women with a history of RVVC, i.e. patients who had had at least four attacks of the condition during the previous year, and who had consulted with symptoms suggesting a new attack of RVVC. The diagnostic criteria applied have been described in detail elsewhere[13,14]. The number of these women who had a positive LE test has already been reported in a previous study[13]. In the present study, we expanded our investigation by comparing the LE test results with findings of candida morphotypes, i.e. of blastoconidia and pseudohyphae, in Gram- and methylene blue-stained vaginal smears. The methods used to identify those with a positive candida culture have been reported in detail elsewhere[15]. In brief, vaginal introital and posterior vaginal fornix samples were cultured on Sabouraud and chromogenic agar[16]. Speciation of isolated candida strains was done with API 50C kits and the Vitek® automised identification system (both from BioMerieu, Paris). The vaginal pH was determined by pH indicator strips (Merck, Darmstadt). The LE activity in vaginal flushing fluid samples was determined by Combur-4® (Roche, Basle) stick.

### Patients attending for control of cytological abnormalities

Twenty-seven women in whom a previous cytological screening had shown cell abnormalities (but no signs of cervical cancer) were investigated. The patients, all of whom were non-pregnant, had no signs or symptoms of VVC or BV. The patients were tested for LE activity, vaginal pH and for presence of inflammatory cells and predominating bacterial flora, i.e. either a predominance of Gram-positive rods (of lactobacilli morphotypes) or a mixture of bacterial morphotypes ('BV flora'). Furthermore, Amsel's criteria were searched for:

**Table 1** Relation between vaginal pH and leukocyte esterase activity in vaginal lavage fluid of women with a history of and an assumed new attack of recurrent vulvovaginal candidiasis

| | | Number of women with indicated test result* | | | |
|---|---|---|---|---|---|
| *Vaginal pH* | *Number of cases* (*n* = 113) | *Grades 0–1* (*n* = 14) | *Grade 2* (*n* = 22) | *Grade 3* (*n* = 77) | *p-value* |
| ≤ 4.4 | 57 | 6 | 13 (12%) | 38 (34%) | 0.904 |
| 4.5–5.4 | 43 | 6 | 7 (6%) | 30 (27%) | 0.903 |
| ≥ 5.5–6.4 | 13 | 2 | 2 | 9 (69%) | 0.927 |

*Grading according to package insert (Combur-4®, Roche, Basle)

increased vaginal pH, a characteristic gray homogeneous vaginal content, presence of clue cells (in methylene blue- and Gram-stained vaginal smears), and a positive amine test obtained by adding 15% KOH to vaginal fluid.

### Patients with vulvovaginal candidosis and bacterial vaginosis and non-pregnant healthy women

Sixteen women with the diagnosis of VVC and 15 with BV were tested for LE activity in vaginal secretion. BV was diagnosed if three of four of Amsel's criteria were fulfilled[4]. The VVC and BV women were all non-pregnant.

The diagnostic methods were the same as those applied in the cases with cytological abnormalities. An acute attack of VVC was considered when the woman reported symptoms such as pruritus, discharge and pain. Signs, such as oedema and erythema of the introitus and the vaginal mucosa with plaques, and/or an increased vaginal discharge (often of cotton-cheese type), were required. The presence of candida blastoconidia and/or hyphae in stained vaginal smears was also required to confirm the diagnosis of VVC.

### Pregnant women

Seventy-three pregnant women were studied. The sampling was made in conjunction with routine maternal check-ups in the second (17–18 weeks of gestation) and third trimester (32–34 weeks of gestation). The diagnostic methods used for the pregnant women were the same as those applied to the women with cytologic abnormalities.

### Statistical analyses

Data were analysed using $\chi^2$ and student *t*-tests. A *p*-value $< 0.05$ was considered significant.

### RESULTS

### Leukocyte esterase activity in cases with history of recurrent vulvovaginal candidosis

In the 113 cases with a history of RVVC and with symptoms suggestive of a new attack of the condition, there was a correlation between the LE activity and the result of the yeast cultures from the genital tract ($p < 0.05$). Thus, of the 63 candida culture-positive women, all had positive LE tests. Of the 50 culture-negative women, 45 had such an increased activity.

There was no correlation between the result of pH determination of vaginal lavage and the result of the LE tests (Table 1). Thus, of 57 women with a vaginal pH of less than 4.5, 51 (89%) had an LE test of grades 2 and 3, while of the 43 women with a pH of 4.5–5.4, 37 (86%) had such a result. Eleven of the 113 women with an even higher pH had a positive LE test (10%).

### Leukocyte esterase activity in correlation to pH in women without vaginitis/vaginosis

Table 2 lists the LE activities in the patients who had no symptoms and/or signs of either VVC or BV, in relation to the vaginal pH. Thus, of the 97 women studied, 76 (78%) had a vaginal secretion pH of 4.7 or less, 45 (59%) of whom had a positive LE test (grades 1–3). Of the 21 women with an elevated vaginal fluid pH (above 4.7), 16 (76%) had a positive LE test. Also in this group, there was no correlation between vaginal pH and LE activity (Table 2).

### Leukocyte esterase activity in vulvovaginal candidosis and bacterial vaginosis and non-vaginitis/vaginosis cases: correlation with occurrence of leukocytes in vaginal lavage fluid

Table 3 shows the LE test results in the VVC, BV, and non-VVC and non-BV cases studied. In 14 (88%) of the 16 VVC cases, the LE activity was elevated (grades 1–3). In the 15 BV cases, this was the case in 11 (73%) patients. In the 97 non-VVC/BV cases, LE activity was elevated in 54 (56%) of the cases investigated. All the VVC cases were symptomatic, as were six of the BV cases. The number of granulocytes in the vaginal smears (less than five, six to nine, or more than ten leukocytes per high-power field, HPF) from the 128 women, is shown in Table 3. There was a correlation between the degree of activity of leukocyte esterase – grades 0, 1, 2 or 3 according to package insert – in the VVC ($p = 0.021$), BV ($p = 0.023$) and in the non-VVC/BV cases ($p = 0.002$).

Table 3 also shows the presence of leukocytes in stained smears of vaginal secretion in relation to LE activity in the vaginitis and vaginosis cases and in women with no signs of these conditions. In 12 (75%) of the 16 VVC cases, and in eight (54%) of the 15 BV patients, there were more than five leukocytes per HPF. In the 97 non-VVC/BV cases, 33 (34%) had such a number of leukocytes in vaginal lavage fluid. There was a correlation between the number of leukocytes and LE activity in the VVC ($p = 0.023$), BV ($p = 0.031$) and non-vaginitis/vaginosis groups ($p = 0.008$).

The positive (PPV) and negative (NPV) predictive values for the LE test (grades 2–3) to foresee an increased number of leukocytes (six or more per HPF) in the patients' stained smears were 53.1 and 77.5%, respectively.

**Table 2** Correlation between grade* of leukocyte esterase (LE) activity in vaginal secretion and pH of the test sample in women without symptoms and clinical signs of vulvovaginal candidosis and bacterial vaginosis

| | | *Number of cases with indicated LE activity* | | | | |
|---|---|---|---|---|---|---|
| *Vaginal pH* | *Total number of women* (*n* = 97) | *Grade* 0 (*n* = 36) | *Grade* 1 (*n* = 17) | *Grade* 2 (*n* = 16) | *Grade* 3 (*n* = 28) | *p-value* |
| ≤ 4.7 | 76 | 31 | 15 | 9 | 21 | 0.07 |
| > 4.7 | 21 | 5 | 2 | 7 | 7 | 0.086 |

*Grading according to package insert (Combur-4®, Roche, Basle)

**Table 3** Correlation between grade* of leukocyte esterase (LE) activity and number of leukocytes in vaginal secretion in women with vulvovaginal candidosis (VVC) and bacterial vaginosis (BV) and in women without evidence of these conditions (controls)

| | | *Number of women with given LE activity and leukocyte count* | | | | | | | | | | | |
|---|---|---|---|---|---|---|---|---|---|---|---|---|---|
| | | *0* | | | *1* | | | *2* | | | *3* | | |
| *Condition* | *Number tested* (*n* = 128) | *A* | *B* | *C* | *A* | *B* | *C* | *A* | *B* | *C* | *A* | *B* | *C* |
| VVC | 16 | 1 | | 1 | | | 2 | | | 2 | 3 | 1 | 6 |
| BV | 15 | 3 | | 1 | | 4 | 1 | 1 | | 8 | 3 | 1 | 1 |
| Controls | 97 | 34 | 3 | 6 | 8 | 1 | 2 | 10 | 2 | 4 | 12 | 4 | 11 |

*Grading according to package insert (Combur-4®, Roche, Basle); A, < 5 leukocytes per high power field (HPF) (x1000); B, 6–9 leukocytes per HPF; C, > 10 leukocytes per HPF

### Leukocyte esterase activity in relation to week in menstrual cycle

Only three of the 64 non-pregnant women were menstruating at the day of examination. LE activity in relation to the start of last menstruation was registered in 53 women. There was no correlation between LE activity and the number of days that had passed since the start of last menstruation (Table 4).

Twelve patients had amenorrhoea, eight of them due to hormonal contraception. The LE test result in this group is also shown in Table 4.

### Leukocyte esterase activity in relation to different trimesters

Of the 73 pregnant women studied, 54 (74%) had an LE activity of grades 1–3 (Table 5). Of the pregnant women, 44 were in the second trimester, while the remaining 29 women had reached the third trimester. In the former group, 32 (73%) and in the latter 22 (76%) women had an increased LE activity in vaginal secretion. In contrast with the non-pregnant women, there was no correlation between the degree of LE activity, neither in the second ($p = 0.75$) nor in the third ($p = 0.74$) trimester.

### Leukocyte esterase activity in relation to predominating bacterial morphotypes in the vagina

In 56 (63%) of the 89 women with a lactobacilli-dominated vaginal flora, independent of the presence of candida morphotypes, LE activity was increased. In 13 (23%) of the 56 women, elongated, non-divided rods of lactobacilli-morphology were detected. In the remaining 43 women with a mixed bacterial flora, increased LE activity was found in 24 (56%). Elongated bacteria were present in six (14%) of the 43 women, three (50%) of whom had elevated LE activity.

### Influence of storage time on leukocyte esterase activity in samples stored in TRIS buffer versus saline

There was no difference in LE activity between vaginal lavage samples collected in TRIS buffer

**Table 4** Leukocyte esterase (LE) activity in relation to the number of weeks since the start of last menstruation

| | | *Number of women with indicated LE activity* | | | |
|---|---|---|---|---|---|
| *Number of weeks since last menstruation* | *Number tested (n = 53)* | *0 (n = 33)* | *1 (n = 7)* | *2–3 (n = 13)* | *p-value* |
| 0–1* | 13 | 10 | 1 | 2 | 0.332 |
| 2 | 12 | 8 | 1 | 3 | 0.335 |
| 3–4** | 28 | 15 | 5 | 8 | 0.090 |
| amenorrhoea | 11 | 5 | 3 | 3 | |

*Ongoing or first week after last menstruation; **or longer

**Table 5** Leukocyte esterase (LE) activity in vaginal lavage of pregnant women in relation to gestational time

| | | *LE activity** | | | | |
|---|---|---|---|---|---|---|
| *Gestational period* | *Number of women tested (n = 73)* | *Grade 0 (n = 19)* | *Grade 1 (n = 8)* | *Grade 2 (n = 17)* | *Grade 3 (n = 29)* | *p-value* |
| Second trimester | 44 | 12 | 6 | 9 | 17 | 0.752 |
| Third trimester | 29 | 7 | 2 | 8 | 12 | 0.742 |

*Grading according to package insert (Combur-4®, Roche, Basle)

or saline. Nor was there any difference in LE activity when analyses were made some minutes after collection compared with the result when the same samples had been stored for up to 6 hours at room temperature.

### DISCUSSION

The determination of LE activity with the aid of different commercial dipsticks has been used to screen for UTI. Some studies have favored their use[9], while others have denied their usefulness for this purpose[5,6].

Although the most common application of LE dipsticks has been in investigations of voided urine for UTI[5,11,12,17,18] or urethritis[7,8], single reports have described their use in tests of vaginal[9] and amniotic fluids[10].

LE tests are often sold in combination with other chemical tests, such as tests for nitrate, hemoglobin and glucose, which all appear on the same dipstick. Evaluation of the value of LE tests for bacteriuria have often been made in the context of concomitant tests for nitrate. However, the specificity for detection of significant bacteriuria, even by use of such a combination of tests, is low[6].

Storage conditions of samples may influence the LE test outcome[18]. Thus, after 24 hours' storage, there is a risk for positive samples to turn negative. In the present study, the tests in the RVVC cases were performed at most a few hours after sampling, while in the other studies, within minutes.

LE tests have been used in screening programs for genital infections of given etiologies, such as chlamydial urethritis and gonorrhoea[7,19–21]. Samples with a positive LE test have then been subjected to a definite etiologic test. Although the sensitivity of LE tests for detection of urethritis in males may be high, the specificity is too low for accurately diagnosing chlamydial and gonococcal infections[7]. In a study of voided urine samples of 224 male military recruits and 443 male sexually transmitted infection clinic patients, we used two enzyme immunoassays, one chemilumminometric assay and an immunofluorescence test to detect *Chamydia trachomatis*. The PPV of LE tests for detection of a genital chlamydial infection was only 44.6%[21].

LE dipstick tests of urine from men with symptomatic trichomoniasis have a higher sensitivity (80%) than specificity (40%)[22]. In the asymptomatic cases of trichomoniasis, the corresponding values were 60 and 68%, respectively. A negative LE test seems to exclude trichomoniasis. In our clinical setting, trichomoniasis is today a rare condition, which was never diagnosed in any of the cases presented in our study.

LE tests have been used to detect asymptomatic bacteriuria in obstetric patients[23,24], and also 'vaginitis of unspecific aetiology' in such patients. Thus, Abbasi and co-workers[9] found LE dipstick tests to have a 100% sensitivity and 90% specificity for detection of such latter cases. They also reported a sensitivity and specificity of 100% for detection of UTI in their patients. In the current study, none of our pregnant women had a recent history of UTI.

BV is generally believed to be an '–osis' and not an '–itis'. In the present study, we used Amsel's criteria[4] to diagnose BV. These criteria do not include any tests for the absence of leukorrhea. However, of our 15 BV patients, seven (47%) had an increased number of leukocytes in vaginal secretion. Furthermore, more than two-thirds (11/15) of the BV cases (defined by Amsel's criteria) had a positive LE test. BV has been considered a risk factor for premature birth, although this is contradicted by several recent prophylactic antibiotic studies[25]. There appears to be a need to recognize the subgroup of pregnant women with BV that seems to be at particular risk to deliver before term. The possibility to use LE tests of vaginal fluid for identifying women at risk should be explored.

The pH of vaginal secretions did not correlate with LE activity in the VVC, BV and non-BV, non-VVC cases. However, some of the women who were diagnosed with BV (i.e. presented with three of the four Amsel's criteria) had a normal vaginal pH, just as some of the VVC cases had an increased pH. These observations contradict the 'text book knowledge' on both vaginosis and vaginitis.

The cycle day might have influenced the LE test result. We found more positive LE tests in the luteal than in the follicular phase, but the difference was not significant. Only three of the patients were menstruating when tested (but note that performance of the LE test is not interfered with by the presence of blood in the test sample).

None of the women were using imipenem, meropenem or clavulanic acid, which all can interfere with the LE test and produce false positive results. Neither had they been prescribed cephalexin or gentamycin, which can diminish the color on the test stick if taken in high doses. In addition, excessive excretion of glucose in urine that contaminates vaginal secretion can have such an influence. In all the pregnant women studied, careful evaluation of the presence of metabolic syndrome had been made as part of the routine maternal check-up at the clinic. Two women with gestational diabetes were included in the study, but they were both metabolically well-controlled.

The PPV of LE dipstick tests for detection of polymorphonuclear leukocytes in urine samples is low[26]. This was also true in the present study of vaginal secretions of women with vaginitis. In RVVC, the vaginal content has often been described as being dominated by monocytes. Our study also showed leukocytes to be present in high numbers in many of the VVC and RVVC cases.

Our study points to the value of examining vaginal smears in the work-up of cases suspected suffering from RVVC[13]. If the diagnosis of this condition is based only on history and clinical examination, approximately half of all such cases will prove negative for *Candida* species[2]. Differentiation between candida-positive and candida-negative cases among women presenting with symptoms and signs generally associated with RVVC is not possible with any high degree of accuracy[14]. The use of one or more doctors' office tests, such as cultures and/or microscopy of stained introital and vaginal smears, is essential in the work-up of cases consulting with genital symptoms. However, our study did not show any benefit from LE determinations by dipstick tests of vaginal secretion in this conjunction.

### REFERENCES

1. Holmes KK, Stamm WE. Lower genital tract syndrome. In Holmes KK, Sparling F, Mårdh P-A, *et al*, eds. *Sexually Transmitted Diseases*. New York: McGraw Hill 1999;761–81
2. Ledger WJ. Current problems in the diagnosis and treatment of candida vaginitis. *Ital J Obst Gyn* 1999;11:25–9
3. Mårdh P-A, Rodrigues AG, Genç M, *et al*. Facts and myths on recurrent vulvovaginal candidosis – a review on epidemiology, clinical manifestations, diagnosis, pathogenesis and therapy. *Int J STD AIDS* 2002;13:522–39
4. Amsel R, Totten PA, Spiegel CA, Chen K, Holmes KK. Nonspecific vaginitis: diagnostic criteria and microbial and epidemiologic associations. *Am J Med* 1983;74:14–22
5. Zaman Z, Borremans A, Verhaegen J, Verbiest L, Blanckaert N. Disappointing dipstick screening of urinary tract infection in hospitalized patients. *J Clin Pathol* 1998;51:471–2
6. Semeniuk H, Church D. Evaluation of the leukocyte esterase and nitrate dipstick screening tests for detection of bacteriuria in women with suspected uncomplicated urinary tract infections. *J Clin Microbiol* 1999;37:3051–2
7. Shafer MA, Schachter J, Moscicki AB, *et al*. Urine leukocyte esterase screening test for asymptomatic chlamydial and gonococcal infections in males. *J Am Med Assoc* 1989;262:2562–6
8. Chernesky M, Jang D, Krepel J, Selleor J, Mahony J. Impact of reference standard sensitivity on accuracy of rapid antigen detection assays and a leukocyte esterase dipstick for diagnosis of *Chlamydia trachomatis* in first-void urine specimens from men. *J Clin Microbiol* 1999;37:2777–8
9. Abbasi IA, Hess LW, Johnson TR, McFadden E, Chernow B. Leukocyte esterase activity in the rapid detection of urinary tract and low genital tract infection in obstetric patients. *Am J Perinatol* 1985;2:311–13
10. Hoskin IA, Katz J, Ordorica SA, Young BK. Esterase activity in second and third trimester amniotic fluid: an indicator of chorioamnionitis. *Am J Obstet Gynecol* 1989;161:1543–5
11. Preston A, O'Donnell T, Phillips CA. Screening for urinary tract infections in a gynaecological setting: validity and cost-effectiveness of reagent strips. *Br J Biomed Sci* 1999;56:253–7
12. Kusumi RK, Grower PJ, Kunin CM. Rapid detection of pyuria by leukocyte esterase activity. *J Am Med Assoc* 1981;245:1653–5

13. Novikova N, Yasseievich E, Mårdh P-A. Microscopy of stained smears of genital secretion in the diagnosis of recurrent vulvovaginal candidosis. *Int J STD AIDS* 2002;13:318–22
14. Novikova N, Mårdh P-A. Characterization of women with a history of recurrent vulvovaginal candidosis. *Acta Obstet Gynecol Scand* 2000;81: 1047–52
15. Novikova N, Rodrigues A, Mårdh P-A. Can the diagnosis of recurrent vulvovaginal candidosis be improved by use of vaginal lavage samples and cultures on chromogenic agar? *Inf Dis Obstet Gynecol* 2002;10:89–92
16. Odds FC, Bernaerts R. CHROMagar Candida, a new differential isolation medium for presumptive identification of clinically important *Candida* species. *J Clin Microbiol* 1994;32:1923–9
17. Gillenwater JY. Detection of urinary leukocytes by Chemstrip. *J Urol* 1981;125:383–4
18. Froom P, Bieganiec B, Ehrenrich Z, Barck M. Stability of common analytes in urine refrigerated for 24 h before automated analysis by test strips. *Clin Chem* 2000;46:1384–6
19. O'Brien SF, Bell TA, Farrow JA. Use of leukocyte esterase dipstick to detect *Chlamydia trachomatis* and *Neisseria gonorrhoeae* urethritis in asymptomatic adolescent male detainees. *Am J Public Health* 1988;78:1583–4
20. McNagny SE, Parker RM, Zenilman JM, Lewis JS. Urinary leukocyte esterase test: a screening method for the detection of asymptomatic chlamydial and gonococcal infection in men. *J Infect Dis* 1992;165:573–6
21. Domeika MA, Basseri M, Mårdh P-A. Non-invasive sampling for detection of genital infection with *Chlamydia trachomatis* in males utilizing urinary leukocyte esterase tests and immuno-assays. *Infection* 1994;22:65–8
22. McNair RD, McDonald SR, Dooley SL, Petterson LR. Evaluation of the centrifuged Gram-stained smear, urinalysis, and reagent strip testing to detect asymptomatic bacteriuria in obstetric patients. *Am J Obstet Gynecol* 2000;182: 1076–9
23. Watson-Jones D, Mugeye K, Mayaud P, *et al.* High prevalence of trichomoniasis in rural men in Mwanza, Tanzania: results from a population-based study. *Sex Trans Dis* 2000;76:355–62
24. Robertson AW, Duff P. The nitrate and leukocyte esterase tests for the evaluation of asymptomatic bacteriuria in obstetric patients. *Obstet Gynecol* 1988;71:878–81
25. Mårdh P-A. Bacterial vaginosis: a treat to reproductive health? Historical perspectives, current knowledge, controversies and research demands. *Eur J Contraception Reprod Health Care* 2000;5:1–12
26. Jacobs JA, De Brauwer EI, Cornelissen EI, Drent M. Correlation of leukocyte esterase detection by reagent strips and the presence of neutrophils: a study in BAL fluid. *Chest* 2000;118:1450–4

RECEIVED 03/14/02; ACCEPTED 09/10/02
